# Toward a Multi-Trait Genetic Panel Targeting Training, Rehabilitation, and Chronic Disease Prevention: A Narrative Review

**DOI:** 10.3390/genes16111309

**Published:** 2025-11-01

**Authors:** Antonio Imperatore, Cristina Mennitti, Giulia De Fonzo, Raffaele Amitrano, Alessandro Gentile, Mariella Calvanese, Fernanda Iafusco, Serena Coppola, Mattia Digno, Paola Borrelli, Barbara Lombardo, Giulia Frisso, Roberto Berni Canani, Nadia Tinto, Valeria D’Argenio, Olga Scudiero

**Affiliations:** 1 Department of Molecular Medicine and Medical Biotechnologies, University of Naples Federico II, Via Sergio Pansini 5, 80131 Naples, Italy; antonio.imperatore2@studenti.unina.it (A.I.); cristina.mennitti@unina.it (C.M.); g.defonzo@studenti.unina.it (G.D.F.); raff.amitrano@studenti.unina.it (R.A.); alessandro.gentile@studenti.unina.it (A.G.); marie.calvanese@studenti.unina.it (M.C.); barbara.lombardo@unina.it (B.L.); gfrisso@unina.it (G.F.); nadia.tinto@unina.it (N.T.); 2 NutriSportHealthLab, University of Naples Federico II, Via Sergio Pansini 5, 80131 Naples, Italy; serena.coppola3@unina.it (S.C.); berni@unina.it (R.B.C.); 3 CEINGE-Biotecnologie Avanzate Franco Salvatore, Via G. Salvatore 486, 80145 Naples, Italy; iafusco@ceinge.unina.it (F.I.); dargenio@ceinge.unina.it (V.D.); 4 Department of Translational Medical Science, University of Naples Federico II, 80131 Naples, Italy; 5 Task Force on Microbiome Studies, University of Naples Federico II, 80100 Naples, Italy; 6 SS Napoli Basket SRL, 80127 Naples, Italy; mattiadigno@gmail.com; 7 Laboratory of Biostatistics, Department of Medical, Oral and Biotechnological Sciences, University G. D’Annunzio of Chieti-Pescara, 66100 Chieti, Italy; paola.borrelli@unich.it; 8 Department of Human Sciences and Quality of Life Promotion, San Raffaele Open University, 00166 Rome, Italy

**Keywords:** genetic polymorphisms, athletic performance, injury susceptibility, personalized training, metabolic risk

## Abstract

Athletic performance results from complex interactions between genetic and environmental factors. This review compiles and synthesizes available literature on polymorphic genes associated with endurance, power, and strength performance, as well as their links to injury susceptibility and chronic metabolic diseases. Endurance performance is modulated by *ACE*, *PPARGC1A*, *HFE*, *UCP2*, *UCP3*, *CDKN1A*, and *PPARA*, regulating mitochondrial biogenesis, oxygen utilization, and muscle fiber composition. Power performance involves *ACTN3*, *MCT1*, *IGF1*, *AMPD1*, *AGT*, and *AGTR2*, affecting anaerobic metabolism, lactate clearance, and fast-twitch fiber recruitment. Strength performance is influenced by *AR*, *PPARG*, *ARK2N*, *MMS22L*, *LRPPRC*, *PHACTR1*, and *MTHFR*, related to androgen signaling, muscle hypertrophy, and recovery. Injury-related genes (*COL1A1*, *COL5A1*, *IL6*, *VEGFA*, *NOG*) and metabolic risk genes (*FTO*, *PPARG*, *ADRB3*) further highlight the clinical relevance of genomics. Collectively, these insights support the application of genetic information to personalize training, enhance performance, prevent injuries, and guide exercise interventions to mitigate metabolic disease risk.

## 1. Introduction

**Figure 1:**
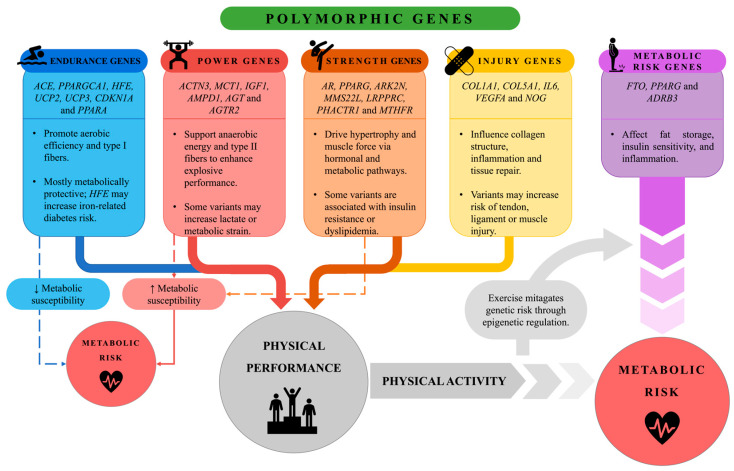
Interplay between polymorphic genes, physical performance, and metabolic risk.

Physical activity is defined as any bodily movement produced by skeletal muscles that involves energy expenditure. Based on the intensity, duration and physiological characteristics involved, it can be classified into endurance (e.g., running, cycling), strength (e.g., weightlifting) and power (e.g., sprinting, jumping) activities [1,2]. Participation in sports or exercise programs is essential for maintaining a healthy lifestyle, with positive impacts both on physical and mental development in different age groups [3]. Furthermore, physical activity plays a key role in the prevention of chronic diseases [3,4]. Sports performance is the result of a complex interaction between environmental and genetic factors. Each discipline has specific physiological, psychological and anthropometric demands that determine a specific athletic phenotype. Numerous studies have shown that athletic success is influenced by genetic traits related to muscle structure, aerobic capacity, strength, and metabolism [5,6,7,8,9,10]. It is estimated that about 66% of the variability in sports performance can be attributed to genetic factors, while the remaining 34% is influenced by environmental elements such as training, nutrition, and medical support [11,12,13]. The heritability of athletic ability varies between 30% and 80%, suggesting the existence of a significant genetic contribution [14]. However, the risk of injury, which is particularly common in young people, must also be considered: it is estimated that approximately 20% of students miss at least one day a year of school due to sports injuries [15,16], while one-third of working adults experience work absences for the same reason [17,18]. Although there is no single “sports gene,” approaches such as the Total Genotype Score (TGS) have shown some potential to discriminate between elite athletes and nonathletes [19]. Moreover, the genetic component does not act in isolation: epigenetic factors can modulate gene expression in response to training and environment, influencing muscle adaptation, recovery capacity, and susceptibility to injury [20]. Understanding the genetic determinants of performance can be useful not only in defining an athlete’s potential within his or her discipline and constructing individualized training programs, but also in guiding exercise prescription in the general population, especially in individuals with a predisposition to metabolic diseases. In recent decades, environmental, economic, and cultural shifts have profoundly influenced the lifestyles, promoting sedentariness and unbalanced diet. These unhealthy habits have resulted in an increased incidence of obesity, insulin resistance, metabolic syndrome, and cardiovascular disease [21]. Obesity, currently affecting about 800 million people worldwide, is defined by the World Health Organization as an excessive accumulation of body fat correlated with increased morbidity and mortality [22]. Excess adiposity induces a chronic inflammatory state with overexpression of pro-inflammatory cytokines, contributing to the genesis of insulin resistance and type 2 diabetes mellitus (T2D) [23]. T2D is a metabolic disorder characterized by altered response to insulin [24]. It is associated with serious complications, including retinopathy, nephropathy, neuropathy, and cardiovascular disease. A crucial parameter in its management is glycated hemoglobin (HbA1c), the increase in which is directly related to the risk of complications. A 1% reduction in HbA1c has been shown to reduce the risk of myocardial infarction by 14% and mortality from diabetes-related causes by more than 20% [25]. The disease is also associated with a systemic inflammatory state with increased leptin, resistin, Tumor Necrosis Factor-alpha (TNF-α) and Interleukin-6 (IL-6), which promote its progression [26]. Formerly considered a disease of adulthood, T2D is now also increasing among young people and children, mainly due to obesity and sedentary lifestyle [27]. In this context, physical activity is an effective strategy in both prevention and management of diabetes [27,28,29] by improving key metabolic markers such as HbA1c, insulin resistance, and fasting insulin [30]. Studies in obese men have shown that aerobic, strength or combination training can reduce insulin resistance and modulate cytokine/adipokine secretion [31]. In at-risk individuals, physical activity has been shown to significantly reduce the likelihood of developing T2D, while in older men a two-month exercise protocol improved insulin sensitivity and fasting blood glucose [32]. In addition, exercise has been observed to promote low-density lipoprotein (LDL) reduction and high-density lipoprotein (HDL) increase, contributing to cardiovascular prevention [33,34,35]. Regular physical activity has been associated with a reduced risk of more than 20 chronic conditions, including cardiovascular disease, stroke, and depression [3,4]. Moreover, it has demonstrated beneficial effects in neurodevelopmental conditions such as autism spectrum disorder (ASD), as well as in neurodegenerative diseases like Alzheimer’s disease (AD) and Parkinson’s disease (PD) [36,37,38]. Given the wide range of positive effects of physical activity on health, an emerging area of research is the exploration of how genetic variability influences individual responses to different types of exercise [39]. Understanding how specific genetic polymorphisms influence physical performance and training adaptations may enable targeted strategies for designing more effective exercise programs both in athletic and clinical populations (Figure 1).

This diagram illustrates the influence of various genetic polymorphisms on physical performance and individual metabolic susceptibility. Endurance-related genes (e.g., *ACE*, *PPARGC1A*, *PPARA*) enhance oxidative metabolism and type I muscle fiber development, contributing to sustained aerobic performance. These variants are generally metabolically protective, although certain alleles, such as those in HFE, may increase the risk of iron-related metabolic disturbances. Power-related genes (e.g., *ACTN3*, *MCT1*, *IGF1*) support anaerobic energy pathways and type II fiber activity, favoring explosive performance. However, some variants may elevate lactate accumulation or cardiometabolic strain. Similarly, Strength-related genes (e.g., *PPARG*, *AR*, *MTHFR*) promote muscle hypertrophy and contractile capacity through hormonal and metabolic regulation. Certain polymorphisms within this group have been associated with adverse metabolic profiles, such as insulin resistance and dyslipidemia, highlighting a potential trade-off between muscular adaptation and metabolic health. Injury-related genes (e.g., *COL1A1*, *IL6*, *VEGFA*) influence connective tissue structure, inflammatory responses, and tissue repair mechanisms. Specific variants can increase susceptibility to muscle, tendon, or ligament injuries, emphasizing the need for personalized load management in training. Additionally, metabolic risk genes (*FTO*, *ADRB3*, *PPARG*) directly affect fat accumulation, insulin sensitivity, and systemic inflammation. Despite genetic predispositions, regular physical activity exerts a beneficial epigenetic effect, modulating gene expression and mitigating metabolic risk. Illustration created by the authors.

Regular physical activity is universally recognized for its broad health benefits, yet individuals differ markedly in their physiological and performance responses to exercise. Such interindividual variability is largely determined by genetic factors that modulate key biological pathways involved in energy metabolism, muscle adaptation, and recovery. Building on this established evidence, the present work aims to provide an overview of the main polymorphic genes associated with the different sports performance phenotypes (endurance, power, and strength) highlighting how genetic assessment can be an essential diagnostic tool for training programs based on the individual genetic profile. An in-depth study of these genetic factors within the general population may offer significant support to clinicians aiming to prescribe personalized exercise interventions based on genetic susceptibility. Finally, the study aims to emphasize how genetic susceptibility to metabolic diseases, such as obesity and type 2 diabetes mellitus, can be effectively counteracted through the adoption of targeted exercise programs and an active lifestyle that can favorably modulate gene expression and improve metabolic outcomes.

## 2. Materials and Methods

This narrative review aimed to synthesize current knowledge on polymorphic genes associated with endurance, power, and strength performance, as well as their links to injury susceptibility and metabolic risk. Relevant studies were identified through PubMed, Scopus, and Google Scholar databases, covering publications up to May 2025. Search terms included combinations of keywords and MeSH terms such as “genetic polymorphisms”, “exercise performance”, “endurance”, “strength”, “power”, “sports injuries”, “metabolic risk”, and “physical activity”. For specific candidate genes, targeted queries were performed using the format “[gene name] polymorphism AND exercise”. 

In order to realize this narrative review, several studies were selected using specific inclusion and exclusion criteria. In particular, inclusion criteria provided original studies in humans, systematic and narrative reviews, and meta-analyses investigating associations between genetic variants and (1) performance-related phenotypes (endurance, strength, power); (2) potential correlation with metabolic risk; (3) susceptibility to sports-related injuries; and (4) predisposition to metabolic risk and its modulation through physical activity.

Instead, exclusion criteria included studies on animal models, case reports, non-peer-reviewed publications, and articles with poorly defined methodology.

Articles were screened by title and abstract, followed by full-text evaluation of potentially relevant studies. Final inclusion was based on thematic relevance to the objectives of this review and the methodological quality reported by the original authors.

Given the narrative nature of this review, no quantitative meta-analysis or formal systematic review tools (e.g., PRISMA, CASP) were applied. The included studies were organized into three thematic domains: (1) genetic variants associated with performance phenotypes (endurance, strength, power) and their potential correlations with metabolic risk; (2) genetic susceptibility to musculoskeletal injuries; and (3) genetic predisposition to metabolic risk and its potential modulation by exercise.

The synthesis aimed to highlight convergent findings, conflicting evidence, and future research directions in the field of exercise genomics.

## 3. Genes Associated with Endurance Performance

Endurance capacity is influenced by a combination of physiological and genetic factors, including muscle fiber composition, hemoglobin mass, mitochondrial biogenesis, maximal cardiac output, and maximal oxygen uptake (VO_2_ max) [40,41,42,43]. Performance in endurance sports, in particular, is largely determined by three key parameters: VO_2_ max, oxygen consumption at the lactate threshold, and movement efficiency [44]. These variables reflect the integration of cardiovascular function, responsible for oxygen transport, and skeletal muscle metabolism, which enables its utilization [45]. Enhanced aerobic endurance has also been linked to increased expression of mitochondrial genes and elevated enzymatic activity involved in aerobic respiration [46]. These intermediate physiological traits display a strong heritable component, with genetic factors estimated to account for up to 70% of their variability [47]. The identification of genetic markers associated with endurance is commonly carried out by comparing allele frequencies between endurance-trained athletes and controls populations. This section focuses on key genetic polymorphisms that have been investigated in relation to endurance athletic performance. Specifically, attention will be given to variants in the angiotensin I converting enzyme (*ACE*), PPARG coactivator 1alpha (*PPARGC1A*), homeostatic iron regulator (*HFE*), uncoupling protein 2 (*UCP2*), uncoupling protein 3 (*UCP3*), cyclin-dependent kinase inhibitor 1A (*CDKN1A*) and peroxisome proliferator-activated receptor alpha (*PPARA*) genes, all of which have been implicated in physiological processes relevant to endurance performance.

### 3.1. Angiotensin I Converting Enzyme (ACE)

Angiotensin-converting enzyme (ACE) is a zinc-dependent metallopeptidase involved in two key physiological processes: the generation of angiotensin II (Ang II) and the degradation of bradykinin. ACE plays a central role in the renin–angiotensin–aldosterone system (RAAS), which regulates blood pressure and electrolyte balance [48]. Specifically, ACE catalyzes the conversion of angiotensin I (Ang I) into Ang II, a potent vasoconstrictor and stimulator of aldosterone secretion, both critical for fluid homeostasis and blood pressure regulation [49]. This system is activated in response to renal hypoperfusion, sodium depletion in the distal tubule, or β-adrenergic stimulation. RAAS has been implicated in various pathophysiological conditions, including hypertension, heart failure, and cardiovascular disease, as well as in physical performance. During exercise, fluid loss leads to decreased plasma volume and blood pressure, which in turn stimulates RAAS activation, promoting sodium and water retention and vascular resistance [50]. The *ACE* gene, located on chromosome 17q23, has been extensively investigated for its role in athletic performance [51]. It exhibits a well-characterized insertion/deletion (I/D) polymorphism (rs1799752), resulting in three genotypes: II (homozygous insertion), ID (heterozygous), and DD (homozygous deletion). The I allele corresponds to the insertion of a 287 bp Alu sequence within intron 16, while the D allele indicates its absence. This polymorphism influences serum and tissue levels of ACE, with significant differences observed across ethnic and sex groups [52,53,54]. The I allele is associated with lower ACE activity and has been consistently linked to enhanced endurance performance [55,56,57], possibly due to improved endothelial function, greater endothelium-dependent vasodilation [58], and a higher proportion of type I (slow-twitch) muscle fibers [59]. Individuals with the II genotype tend to exhibit elevated VO_2_ max and improved cardiorespiratory efficiency [60,61]. Consequently, ACE activity may influence not only vascular remodeling but also mitochondrial efficiency and the individual response to aerobic training. Numerous studies have reported a higher prevalence of the type II genotype among elite endurance athletes across a range of disciplines, including mountaineering, rowing, distance running, cycling, triathlon, and handball [56,57,62,63,64,65,66,67]. The functional advantage conferred by the I allele appears to stem from its capacity to attenuate ACE expression, thereby reducing Ang II levels and enhancing skeletal muscle perfusion [68,69]. Moreover, decreased ACE activity is associated with increased nitric oxide (NO) bioavailability in skeletal muscle, which may promote mitochondrial efficiency and contractile function, especially during high-intensity exercise [70,71]. Conversely, the D allele is correlated with higher circulating and tissue ACE levels [72], and has been associated with superior muscular strength and power, favoring anaerobic performance [55]. A higher frequency of the D allele has been observed in elite power athletes, including British, Russian, and European swimmers [57,65,73]. The elevated ACE activity in D allele carriers enhances the conversion of Ang I to Ang II [74], potentially supporting rapid muscle activation and hypertrophy. Thus, while the II genotype may improve muscle mechanical efficiency via a higher prevalence of slow-twitch fibers [75], D allele carriers may benefit from greater muscle power output and a predominance of fast-twitch fibers [76,77]. Given ACE’s centrality within the RAAS, the I/D polymorphism has also been investigated in relation to microvascular complications and metabolic disorders. Dysregulation of the RAAS has been implicated in insulin resistance and type 2 diabetes mellitus (T2D) [78], and pharmacological blockade of the RAAS can mitigate T2D-related complications [79,80]. However, studies examining the association between the *ACE* I/D polymorphism and T2D risk have yielded inconsistent results. While some studies report a higher prevalence of the D allele among T2D patients [81,82], others have found no significant association between this variant and disease susceptibility [83].

### 3.2. PPARG Coactivator 1alpha (PPARGC1A)

The PPARG coactivator 1alpha (PPARGC1A) gene encodes the transcriptional coactivator PGC-1α, a member of the peroxisome proliferator-activated receptor (PPAR) family [84]. PGC-1α plays a pivotal role in the regulation of numerous metabolic pathways, including fatty acid oxidation, glucose utilization, thermogenesis, and angiogenesis [84,85]. It also promotes mitochondrial biogenesis through the activation of nuclear respiratory factors, NRF1 and NRF2, as well as the expression of mitochondrial transcription factor A (TFAM) [86]. Overexpression of PGC-1α has been shown to induce a shift in skeletal muscle fiber composition towards type I fibers, which are mitochondria-rich and characterized by high oxidative capacity and fatigue resistance. This effect is mediated through the coactivation of myocyte enhancer factor 2 (MEF2) [87]. Located on chromosome 4p15.2, the PPARGC1A gene harbors a common non-synonymous single-nucleotide polymorphism (SNP), rs8192678 (G/A) (Gly482Ser), which results in an amino acid substitution from glycine (Gly) to serine (Ser). This variant has been associated with athletic performance, with the Gly/Gly genotype linked to greater oxidative capacity, higher mitochondrial content, and enhanced fatigue resistance [88]. Several studies in Caucasian endurance athletes have reported a lower frequency of the Ser482 allele, which has been associated with reduced aerobic capacity [89,90,91]. Conversely, the Gly482 allele is considered a favorable genetic factor for aerobic metabolism and endurance performance [90,92]. Nevertheless, some research indicates that Ser482 carriers may exhibit improved VO_2_ max response to training [93], and the Ser/Ser genotype has been observed at a higher frequency among powerlifters, suggesting a potential role of this variant in strength-related phenotypes as well [94]. Beyond athletic performance, the Gly482Ser polymorphism has been implicated in susceptibility to obesity, type 2 diabetes mellitus (T2D), and hypertension [95,96,97]. However, the association appears to vary across populations. The Ser482 allele has been associated with increased T2D risk in populations from West and South Asia, Europe, and Africa, but no significant association has been observed in East Asian cohorts [98]. Lifestyle factors, such as physical activity levels and body composition, may influence the phenotypic expression of this polymorphism, particularly regarding parameters of insulin resistance [99].

### 3.3. Homeostatic Iron Regulator (HFE)

Iron is an essential trace element, critically involved in oxygen transport and storage due to its incorporation into key proteins such as hemoglobin (Hb), the main oxygen carrier in blood, and myoglobin, which facilitates oxygen accumulation and transfer to mitochondria in active skeletal muscle [100]. Iron also plays a central role in erythropoiesis, the process of red blood cell (RBC) production, thus ensuring adequate oxygen delivery to tissues, including skeletal muscle and the heart [100]. Iron homeostasis is tightly regulated at the systemic level by multiple genes, including homeostatic iron regulator (*HFE*), which encodes a membrane protein involved in the control of dietary iron absorption and systemic distribution [101]. The HFE protein interacts with transferrin receptor 2 (TFR2) and modulates the expression of hepcidin, a liver-derived peptide hormone that inhibits intestinal iron absorption by binding to and promoting degradation of ferroportin, the main iron exporter from enterocytes to the bloodstream [101,102]. Two SNPs in the *HFE* gene, Cys282Tyr rs1800562 (G/A) and His63Tyr rs1799945 (C/T), have a significant impact on HFE protein function and iron metabolism regulation. The C282Y variant (845G > A) alters the protein’s structure, impairing its interaction with TFR2 and disrupting hepcidin regulation [101]. Reduced hepcidin levels lead to increased ferroportin activity, enhancing intestinal iron absorption and promoting systemic iron overload, which can lead to oxidative stress and tissue damage [101,103]. Individuals homozygous for the A allele (C282Y/C282Y) are at high risk of developing hereditary hemochromatosis (HH), a disorder characterized by pathological iron accumulation in various tissues [104,105]. The H63D polymorphism rs1799945 (C/G) leads to a histidine-to-aspartic acid substitution in the HFE protein, reducing its binding affinity for TFR2 and causing a moderate decrease in hepcidin expression, although less severe than that observed with C282Y variant [106]. The G allele (63D) has been linked to elevated iron stores, although the risk of HH associated with this variant is considerably lower than that of C282Y [102]. Epidemiological studies have reported a higher prevalence of *HFE* risk alleles among elite athletes compared to the general population [107,108]. Hermine et al. found that 41% of French elite athletes and 80% of medalists in European/international competitions carried at least one *HFE* risk variant, compared to 27% in the general population [107]. The reduced hepcidin expression observed in carriers may increase iron availability for erythropoiesis, enhancing Hb production and oxygen transport, thus improving aerobic capacity, a key determinant of endurance performance [102,107,109,110]. Further support comes from genome-wide association studies (GWAS), which have identified a significant association between the H63D variant and various hematological parameters, including hematocrit, mean corpuscular hemoglobin concentration, and reticulocyte count, suggesting a potential contribution to aerobic endurance [111,112]. Consistent with these findings, Thakkar et al. reported that athletes with intermediate or high-risk *HFE* genotypes exhibited 17% higher VO_2_ peak values compared to those with low-risk genotypes, highlighting the genetic influence on aerobic performance [113]. However, these advantages may come at a metabolic cost. Some evidence suggests an association between the H63D polymorphism and an increased risk of type 2 diabetes mellitus (T2D), likely due to iron-mediated oxidative stress and pancreatic beta-cell dysfunction, impairing glycemic regulation [114]. Several studies have reported a modestly elevated T2D risk among H63D carriers compared to non-carriers [115,116], while the C282Y variant has not been consistently associated with T2D development [115]. Taken together, these findings suggest that although *HFE* polymorphisms may confer aerobic performance benefits in athletes, they may also predispose individuals to metabolic disturbances later in life.

### 3.4. Uncoupling Protein 2 (UCP2) and Uncoupling Protein 3 (UCP3)

Uncoupling protein 2 (UCP2), a member of the mitochondrial anion carrier protein (MCAP) family, is expressed across various tissues, including skeletal muscle, myocardium, kidneys, lungs, spleen, central nervous system, and white adipose tissue [117]. In adipose tissue, the partial decoupling between electron transport and oxidative phosphorylation leads to proton leakage mediated by UCPs, thereby dissipating energy as heat and reducing mitochondrial efficiency [118]. Although the exact physiological role of UCP2 remains to be fully defined, aerobic training has been shown to upregulate its expression in skeletal muscle and cardiac tissue [118]. From a genetic standpoint, the Ala55Val rs660339 (C/T) polymorphism in the *UCP2* gene has been associated with the Val allele conferring enhanced maximal oxygen uptake (VO_2_ max), improved exercise efficiency [119], increased physical activity and metabolic efficiency [120], as well as a predisposition to endurance performance [121]. Gronek et al. reported an overrepresentation of the Val allele among high-level runners, suggesting a possible association between the CT genotype and half-marathon performance [122]. In contrast, Sessa et al. observed a higher frequency of the Ala allele in athletes focused on power-based disciplines [123]. Given the close genomic proximity (~8 kb) of the *UCP2* and *UCP3* genes on chromosome 11q13, Buemann et al. proposed that the observed associations with metabolic and exercise-related traits may reflect linkage disequilibrium with a functional variant within *UCP3* [119]. Uncoupling protein 3 (UCP3), another mitochondrial uncoupling protein, is predominantly expressed in skeletal muscle and brown adipose tissue [124], where it contributes to the reduction in mitochondrial reactive oxygen species (ROS) production, potentially mitigating endothelial oxidative stress [124,125]. The -55C/T polymorphism in the *UCP3* promoter has been shown to increase gene expression and resting energy expenditure, thereby enhancing aerobic potential and reducing obesity risk [124,126]. Numerous studies have investigated the roles of *UCP2* (Ala55Val) and *UCP3* (-55C/T) variants in relation to obesity, lipid metabolism, and type 2 diabetes mellitus (T2D), with often inconsistent findings [127]. Specifically, individuals homozygous for the Val allele of *UCP2* Ala55Val exhibited reduced mitochondrial uncoupling, increased metabolic rate, and a higher risk for obesity and T2D [128,129]. Conversely, in other cohorts, the same genotype was associated with greater weight loss and elevated BMI values [130]. Other investigations failed to demonstrate any significant associations between Ala55Val and basal metabolic rate, metabolic syndrome, BMI, insulin secretion, or T2D [131,132,133]. Regarding *UCP3*, the -55T allele has been linked to lower BMI and higher HDL cholesterol levels [125,134], potentially due to enhanced mRNA expression leading to increased lipid oxidation [134]. However, alternative studies associated the C/T genotype with lower *UCP3* mRNA levels [135] and a reduced risk of obesity [136]. On the contrary, some evidence links the T allele to increased BMI [137] and waist circumference [138]. Several studies reported no significant associations between the -55C/T polymorphism and metabolic traits such as resting metabolic rate, insulin secretion, obesity, or T2D [139,140].

### 3.5. Cyclin-Dependent Kinase Inhibitor 1A (CDKN1A)

The cyclin-dependent kinase inhibitor 1A (*CDKN1A*) gene encodes p21, a multifunctional regulator involved in several fundamental cellular processes, including cell cycle control, stem cell proliferation, transcriptional regulation, apoptosis, DNA repair, and cell motility [141]. These functions are mediated by p21 through its interactions with multiple key proteins involved in these biological pathways [142]. Conversely, miR-208b negatively regulates *CDKN1A* expression by binding to its 3′-untranslated region (3′-UTR), thereby significantly influencing the proliferation and differentiation of skeletal muscle cells [143]. In addition to its regulatory role, miR-208b is critically involved in muscle fiber specification, promoting the slow-twitch phenotype and modulating AMPK/PGC-1α signaling, a key pathway in mitochondrial biogenesis [144,145]. In this context, *CDKN1A* has been identified as a genetic determinant of muscle fiber composition and athletic predisposition. Specifically, the *CDKN1A* SNP rs236448 (A/C) has been associated with the proportion of slow-twitch fibers, where the A allele has been linked to enhanced endurance capacity and superior performance in aerobic-based sports [146]. Human skeletal muscle is composed by three major fiber types: type I fibers (slow-twitch, oxidative), type IIA fibers (fast-twitch, oxidative), and type IIX fibers (fast-twitch, glycolytic), each with distinct functional characteristics. Type I fibers, characterized by high fatigue resistance, are typically enriched in endurance-trained athletes [10,147,148]. The distribution of muscle fiber types is influenced by both genetic and environmental factors and carries significant health implications. A lower percentage of type I fibers has been associated with an increased risk of obesity, insulin resistance, and hypertension [10,41,149]. Therefore, the regulatory relationship between *CDKN1A* and miR-208b not only influences muscle fiber composition but may also serve as a potential target for performance optimization and therapeutic intervention in metabolic and neuromuscular disorders.

### 3.6. Peroxisome Proliferator-Activated Receptor Alpha (PPARA)

The peroxisome proliferator-activated receptor alpha (*PPARA*) gene, located on chromosome 22q12–q13.1, encodes PPARα, a nuclear transcription factor involved in the regulation of lipid metabolism and energy homeostasis [150]. This receptor is activated under conditions of energy deprivation and metabolic or physiological stress, including physical exercise, and plays a key role in maintaining energy balance. It regulates the uptake and utilization of fatty acids and glucose, particularly in metabolically active tissues such as the liver, heart, and skeletal muscle [151,152,153]. PPARα modulates the transcription of several genes involved in mitochondrial β-oxidation, including acyl-CoA oxidase, thereby influencing energy substrates selection, such as the shift between glucose and fatty acids, during exercise and contributing to cardiac function and adaptation [154,155,156]. Additionally, PPARα is involved in mediating inflammatory responses and regulating vascular function [157]. Among the genetic variants of *PPARA*, the most extensively studied is a G > C substitution rs4253778 (G/C) located in intron 7. The G allele has been associated with increased PPARα expression, a higher proportion of slow-twitch (type I) muscle fibers, and improved lipid oxidation efficiency [154,155]. These muscle fibers, commonly observed in endurance athletes, exhibit greater oxygen utilization during prolonged activity, suggesting a potential advantage for aerobic performance [121,158]. Specifically, the GG genotype has been linked to higher maximal aerobic capacity and enhanced oxygen pulse [159]. Studies by Eynon et al. [89] and Ahmetov et al. [121] have also reported a higher frequency of the G allele in endurance athletes compared to those engaged in sprint or power disciplines. However, the literature presents some inconsistencies. Certain investigations found no significant association between the GG genotype and endurance performance in the general, untrained population [160]. Conversely, the C allele has been linked to a greater prevalence of fast-twitch (type II) muscle fibers, which are suited for rapid and forceful contractions, potentially conferring an advantage in strength- and power-oriented sports [161]. Supporting this, Ginevičienė et al. observed that Lithuanian athletes carrying the CC or GC genotypes exhibited greater lower-limb muscle mass and strength compared to GG homozygotes [162]. Similarly, Végh et al. suggested that the CC genotype may confer performance benefits under prolonged training, promoting long-term metabolic adaptations to physical effort [163]. From a metabolic perspective, rs4253778 has also been associated with dyslipidemia and increased cardiovascular risk. The C allele, in particular, has been linked to increased serum total cholesterol (TC) and low-density lipoprotein (LDL) levels, indicating a predisposition to lipid metabolism disorders [164]. Flavell et al. reported more pronounced atherosclerosis progression in C allele carriers compared to GG homozygotes [165], and Doney et al. observed a higher incidence of myocardial infarction associated with the C allele in a Scottish cohort [166]. Furthermore, the C allele has been associated with elevated levels of fetuin-A, a liver-derived glycoprotein involved in obesity development [167]. Although rs4253778 (G/T) is located in an intronic region and thus theoretically non-coding, evidence suggests it may influence gene expression, lipid metabolism, muscle fiber composition, and responses to physical activity. These characteristics make *PPARA* a compelling candidate gene for both athletic performance profiling and the prevention and management of metabolic risk.

## 4. Genes Associated with Power Performance

With the advancement of molecular research in sports science, several genes associated with power, strength, and endurance have been identified. Current investigations focus not only on how genetic makeup influences athletic performance [5,168], but also on how environmental factors and training can, in some cases, modulate the expression of specific genes [6,169]. Genes related to power-athlete status play a pivotal role in regulating physical performance, particularly by affecting the capacity to generate muscular power. Polymorphisms in these genes may account for individual variability in exercise responses, with significant implications for athletic outcomes [170,171]. Moreover, certain genetic variants have been linked to an increased susceptibility to metabolic disorders, highlighting a complex interplay between genetics, training, and metabolic health [172]. Notably, skeletal muscle alone contributes to approximately 30% of basal metabolic rate, even at rest [173]. This section focuses on key genetic polymorphisms that have been investigated in relation to power athletic performance. Specifically, attention will be given to variants in the actinin alpha 3 (*ACTN3*), solute carrier family 16 member 1 (*SLC16A1/MCT1*), insulin-like growth factor 1 (*IGF-1*), adenosine monophosphate deaminase 1 (*AMPD1*), angiotensinogen (*AGT*), and angiotensin II receptor type 2 (*AGTR2*) genes. These polymorphisms have been associated with traits such as muscle fiber composition, energy metabolism, and cardiovascular regulation, all of which are critical determinants of performance in power-oriented sports.

### 4.1. Actinin Alpha 3 (ACTN3)

The actinin alpha 3 (*ACTN3*) gene encodes α-actinin-3, a structural protein predominantly expressed in type II fast-twitch muscle fibers, which are responsible for generating explosive power and strength. Due to its specific role in muscle function, *ACTN3* is regarded as a key gene influencing power and sprint performance [174]. Among the numerous polymorphisms investigated in elite athletic populations, the R577X variant (rs1815739) stands out for its consistent association with performance in sprint and power sports. It remains one of the few polymorphisms repeatedly linked to athletic status across diverse elite cohorts [175], though findings are often limited by small sample sizes. R577X is a nonsense single-nucleotide polymorphism (SNP) that introduces a premature stop codon, impairing the production of functional α-actinin-3 in individuals homozygous for the X allele (XX genotype). While those with RR or RX genotypes express the protein normally, XX individuals lack α-actinin-3 entirely [176]. However, this deficiency does not lead to overt muscular dysfunction due to a compensatory upregulation of α-actinin-2 [177]. A meta-analysis by El Ouali et al. compared the R577X genotype distribution in power athletes, endurance athletes, and non-athletic controls. The findings revealed a significantly higher frequency of the RR genotype in power athletes, and a relative underrepresentation of the XX genotype, supporting the hypothesis that the R allele confers an advantage in power-oriented sports [178]. Interestingly, the RR genotype has also been associated with higher testosterone levels [179], which may partly explain its link to enhanced muscle hypertrophy and power-based athletic status [180]. Nonetheless, inconsistent findings have been reported. Some studies, such as those conducted on Lithuanian and Russian athletes engaged in weightlifting and throwing disciplines, found no significant differences in R577X genotype distributions [94]. Similarly, a study by Demirci et al. involving 101 elite basketball players observed a lower frequency of the RR genotype in athletes compared to controls, while acknowledging that limited sample size likely reduced statistical power [181]. For instance, a study by Ben-Zaken et al. on swimmers revealed no significant difference in genotype distribution between short-distance (power) and long-distance (endurance) athletes, suggesting that factors beyond genetics, such as technique and psychological resilience, are critical to success in certain sports [182]. Beyond athletic performance, the *ACTN3* R577X variant has also been associated with metabolic health markers. Individuals with the XX genotype exhibit elevated blood glucose, triglycerides, and total cholesterol, along with reduced levels of HDL cholesterol, a key factor in cardiovascular protection [183]. Although the XX genotype is more prevalent among individuals with type 2 diabetes, it does not appear to significantly impact glycemic control or obesity status [184].

### 4.2. Solute Carrier Family 16 Member 1 (SLC16A1/MCT1)

The solute carrier family 16 member 1 (*SLC16A1*, also known as *MCT1*) gene encodes the Monocarboxylate Transporter 1(MCT1), a transmembrane protein responsible for the transport of lactate and other monocarboxylates across cell membranes [185]. MCT1 is essential for maintaining lactate homeostasis, especially during physical exertion, by regulating both its influx and efflux in skeletal, cardiac, and cerebral tissues [186,187]. During high-intensity exercise, when lactate is produced as a by-product of anaerobic glycolysis, MCT1 supports its reutilization for energy production within mitochondria, highlighting its central role in energy metabolism [188]. The gene is relevant for both endurance and power athletes. Higher MCT1 expression has been observed in endurance athletes, supporting greater lactate clearance and utilization. Conversely, power athletes may benefit from genetic variants that enhance the removal of lactate following anaerobic exertion, thus helping to delay fatigue [189]. A well-characterized variant, rs1049434 (A/T), involves a single-nucleotide substitution resulting in an amino acid change from lysine to methionine [190]. This polymorphism alters cellular energy metabolism. Individuals with the AA genotype exhibit greater glycogen depletion and elevated NADH levels, suggesting reduced pyruvate-to-lactate conversion. In contrast, T allele carriers (AT/TT) demonstrate increased baseline lactate accumulation, favoring anaerobic energy production [191]. This may be disadvantageous in endurance sports but beneficial in power disciplines, where energy is required over shorter durations [192]. However, it should be noted that allele nomenclature for rs1049434 has varied across studies: according to the 1000 Genomes database, the more frequent, normally functioning allele is T, whereas the less frequent allele A is associated with higher lactate levels and is underrepresented in endurance athletes. Early studies sometimes labeled the more frequent allele as A, which may cause confusion. Although the mechanisms remain partially unresolved, the T allele has been linked to enhanced athletic performance, initially suggesting its association with endurance capacity [193]. However, more recent meta-analyses classify it as a power-related allele, with the TT genotype frequently identified among elite power athletes [194]. For example, Pasqualetti et al. found that rugby players with the TT genotype showed superior peak vertical power output, whereas those with the AA genotype demonstrated better speed and agility, further emphasizing MCT1’s multifaceted role in sport-specific traits [195]. Interestingly, MCT1 expression may be modulated by training intensity, particularly under intermittent hypoxic conditions [196]. Hypoxic training has been shown to enhance oxygen transport capacity through increased red blood cell mass and hemoglobin concentration [197]. In addition, prolonged anaerobic exercise induces buffering adaptations to counteract acidosis by promoting H+ ion clearance via MCT1 [198]. These observations have led to the development of intermittent hypoxic training (IHT) protocols, which have improve peak power output without altering VO_2_ max [199,200,201,202,203]. Though IHT appears to influence lactate metabolism, Millet et al. reported no significant changes in MCT1 expression post-training, highlighting the need for further research [204,205]. Beyond athletic performance, MCT1 has been implicated in glucose metabolism and the pathophysiology of type 2 diabetes (T2D). Genetic variants may influence lactate and alanine processing, affecting pancreatic islet function [206]. Elevated MCT1 expression in pancreatic tissue has been associated with dysregulated insulin secretion, potentially contributing to certain forms of T2D [207].

### 4.3. Insulin-like Growth Factor 1 (IGF1)

The insulin-like growth factor 1 (*IGF1*) gene encodes IGF1, a peptide hormone structurally related to insulin, that plays a pivotal role in muscle development, regeneration, and energy metabolism, and is secreted in response to growth hormone (GH) released by the hypothalamus. It is involved in several physiological processes relevant to athletic performance [208]. One of IGF1 primary roles is to stimulate muscle hypertrophy, through the activation of the PI3K-AKT-mTOR pathway, which promotes protein synthesis and muscle cell growth, leading to increased muscle mass [209]. Additionally, IGF1 supports muscle regeneration and recovery, by stimulating the proliferation of satellite cells that repair muscle fibers post-exercise. It also reduces muscle catabolism by counteracting myostatin and other catabolic myokines [210]. IGF1 also contributes to strength and power performance by promoting the development of type II muscle fibers, which are essential for explosive power, and by improving bone density and tendon resilience, both of which are important factors in athletic performance [211,212]. Moreover, IGF1 plays a role in energy metabolism by enhancing glucose uptake and insulin sensitivity in muscle tissue, thereby optimizing energy utilization during physical exertion [213]. Collectively, these functions make IGF1 a central mediator of muscle strength, hypertrophy, and recovery, particularly important for athletes requiring explosive power. The anabolic role of endogenous IGF1 has been specifically noted in female athletes involved in power sports [214]. A polymorphism in the promoter region of the *IGF1* gene, rs35767 (C/T), has been identified as a regulator of circulating IGF1 levels and may influence both power and endurance performance [215]. However, literature data remain inconsistent, and no clear distribution pattern has been established [216,217]. Nevertheless, the T allele is more frequently observed in power athletes, whereas the C/C genotype has been associated with lower muscle mass and higher body fat percentage, possibly impairing performance in strength-based sports [216,217,218,219]. IGF1 is also involved in glucose and lipid metabolism, playing a role in glucose homeostasis and promoting fatty acid β-oxidation during fasting, thereby reducing circulating lipid levels [220,221,222,223]. These effects have prompted investigations into its role in type 2 diabetes (T2D). Several case–control studies suggested an association between rs35767 and T2D risk [224,225]. However, a meta-analysis by Zeng et al. concluded that there is no statistically significant association between *IGF1* polymorphisms and the development of T2D [226]. In summary, while IGF1 is essential for muscle growth and metabolic regulation, the functional role of rs35767 remains unclear, particularly in relation to athletic predisposition and disease risk.

### 4.4. Adenosine Monophosphate Deaminase 1 (AMPD1)

The adenosine monophosphate deaminase 1 (*AMPD1*) gene encodes AMP deaminase 1, a key enzyme involved in the energy metabolism of skeletal muscle. This enzyme catalyzes the conversion of adenosine monophosphate (AMP), a byproduct of ATP consumption, into inosine monophosphate (IMP) [227]. This reaction is part of the purine nucleotide cycle [228], which contributes to maintaining cellular energy homeostasis during short bursts of high-intensity exercise, where energy demands are particularly elevated. As the muscle-specific isoform, AMPD1 is highly expressed in fast-twitch (type II) muscle fibers, making it especially relevant in power-oriented sports, such as sprinting, weightlifting, and combat disciplines, where rapid ATP regeneration is crucial [229]. Furthermore, AMPD1 expression appears to be modulated by exercise intensity [230]. Variations in gene expression across different muscle fiber types may contribute to differences in enzyme activity between individuals. One of the most well-characterized polymorphisms within the *AMPD1* gene is rs17602729 (C/T). This nonsense mutation (c.34C > T) occurs in exon 2 and leads to a premature stop codon, resulting in partial or complete absence of the functional enzyme [231]. Individuals carrying the T allele, especially those homozygous for it genotype (TT), often exhibit AMPD1 deficiency, which can manifest as early muscle fatigue, cramping, or reduced tolerance to anaerobic exercise [232]. Conversely, individuals with the CC homozygotes genotype typically exhibit full enzyme activity and are more predisposed to excel in anaerobic, high-intensity physical tasks [233]. Beyond its role in sports performance, *AMPD1* polymorphisms have also been linked to metabolic outcomes, particularly in relation to insulin clearance. Specific haplotypes within the gene appear to influence interindividual variability in insulin metabolism [234]. Interestingly, the C34T variant has been associated with a lower risk of obesity among patients with coronary artery disease (CAD). Moreover, this variant has been suggested to reduce the likelihood of hyperglycemia and type 2 diabetes, even outside of CAD contexts [235]. Despite its physiological relevance, the T allele is relatively uncommon in the general population, with a heterozygous frequency of around 10% in Europe, and the TT genotype occurring in only 2% of individuals [236]. A study conducted by Safranow et al. examined *AMPD1* mutations in patients with CAD or heart failure, finding that the C34T mutation correlated with a lower prevalence of diabetes and obesity [237]. In contrast, a rarer polymorphism in exon 7, A860T, appears to have opposite metabolic effects [238].

### 4.5. Angiotensinogen (AGT)

The angiotensinogen (*AGT*) gene encodes angiotensinogen, a globular glycoprotein that serves as a critical precursor in the renin–angiotensin–aldosterone system (RAAS). Synthesized primarily in the liver, angiotensinogen is cleaved by renin to form angiotensin I, which is subsequently converted into angiotensin II (Ang II) by the angiotensin-converting enzyme (ACE) [239]. Ang II increases blood pressure, promotes sodium and water retention, and contributes to inflammation and fibrosis, thereby accelerating the progression of cardiovascular and renal diseases [240]. Located on chromosome 1q42 [241], *AGT* is characterized by several polymorphisms, the most widely studied being the M235T variant rs699 (T/C). This missense polymorphism results in the substitution of methionine with threonine at residue 235, leading to a 10–30% increase in plasma AGT levels among carriers of the C allele [242]. Elevated AGT is associated with higher levels of Ang II, which also functions as a skeletal muscle growth factor, potentially conferring a performance advantage in power athletes [243,244]. González-Estrada et al. reported a significantly higher frequency of the C allele among elite athletes compared to controls, suggesting a potential link to strength-related performance rather than endurance [245]. Ethnic variation in the allele distribution of M235T has been reported, with the T allele being more prevalent among African, African American, and Japanese populations [241,246]. Beyond its cardiovascular role, AGT affects several physiological responses to exercise, including blood pressure regulation, cardiorespiratory fitness, and cardiac morphology [246,247,248,249]. Moreover, the RAAS plays a role in glucose metabolism by modulating insulin secretion and sensitivity [250]. RAAS activation in skeletal muscle, adipose tissue, and the pancreas may lead to insulin resistance in genetically susceptible individuals [251]. Polymorphisms in AGT have been associated with metabolic syndrome, type 2 diabetes mellitus (T2D), hypertension, and altered insulin sensitivity [252]. AGT is also secreted by adipocytes and acts as a cytokine [253]. In Japanese obese women, the CC genotype of M235T has been associated with visceral adiposity and hyperinsulinemia [254], while the T allele appears to be associated with an increased risk of developing T2D [255]. Although AGT plasma levels are relatively stable within individuals, they can be modulated by hormones such as glucocorticoids, estrogens, thyroid hormones, and Ang II itself [256]. Genetic interactions among AGT, ACE, and Angiotensin II Type 1 Receptor (AT1R), which are part of the same metabolic pathway, can influence the synthesis of their respective end-products and collectively elevate the risk of T2D and other disorders. This risk is modulated by the population-specific frequency of these alleles [255,257].

### 4.6. Angiotensin II Receptor Type 2 (AGTR2)

The angiotensin II receptor type 2 (*AGTR2*) gene encode the type-2 angiotensin II receptor (AT2R), a component of the renin–angiotensin–aldosterone system (RAAS). In contrast to the type-1 angiotensin II receptor (AT1R), which mediates vasoconstrictive and pro-inflammatory effects, AT2R exerts opposing actions by promoting vasodilation, anti-inflammatory and antifibrotic responses, neuroprotection, and regulation of cell growth. This receptor is expressed in various tissues, including the heart, kidneys, brain, and blood vessels, playing a protective role against cardiovascular diseases. Additionally, it is considered a regulator of skeletal muscle growth and differentiation, being associated with the composition of type 2 muscle fibers and aerobic physical activity [243,258,259]. Mustafina et al. have demonstrated that polymorphisms in the *AGTR2* gene are associated with training quality and sports performance [260]. Specifically, the A allele of the rs11091046 (C/A) polymorphism has been linked to a higher percentage of fast-twitch fibers and disciplines focused on power, while the C allele appears to be associated with a greater proportion of slow-twitch muscle fibers, suggesting a correlation with endurance athlete status and aerobic performance [260]. However, subsequent studies have reported conflicting results. For instance, Yvert et al. observed a higher frequency of the C allele in male sprint/power athletes in Japanese and Polish–Russian cohorts, suggesting that the C allele may be favorable for sprint/power performance in men [261]. This discrepancy could stem from the inclusion of various sports disciplines in Mustafina et al.’s study, making it challenging to draw definitive conclusions. Both studies agree that the C allele is unfavorable in women concerning sprint/power activities [260,261]. Gender differences in results may be attributed to skewed X-chromosome inactivation, as the *AGTR2* gene is located on this chromosome, and the presence of women with random X inactivation could have influenced the findings [262]. Additionally, there may be gender-specific differences in the regulation of the RAAS [263]. Despite the conflicting results, it remains beneficial to study this gene, potentially exploring interactions with other genes, as angiotensin II, through the AT1R and AT2R, plays a crucial role in regulating vascular tone and promoting muscle growth. Genotypes influence the type and proportion of muscle fibers, and understanding these variations can aid in optimizing training programs for athletes [194].

## 5. Genes Associated with Strength Performance

Performance in strength-based sports arises from the complex interplay between various physiological and genetic factors that influence an athlete’s ability to generate muscular force effectively. Key contributors to strength, phenotypes include muscle hypertrophy (fiber size increase), hyperplasia (fiber number increase), a predominance of fast-twitch fibers, optimized neural adaptations, high glycolytic capacity, and elevated circulating testosterone levels [264]. Substantial evidence indicates that strength athletes exhibit significant differences compared to both endurance athletes and untrained individuals in terms of transcriptomic, biochemical, anthropometric, physiological, and biomechanical traits [6]. These distinctions are shaped by both environmental inputs, such as training and diet, and a strong genetic component. In fact, muscular strength traits have been found to be highly heritable, with genetic factors accounting for up to 85% of the variability in maximal strength measured through isometric, isotonic, and isokinetic methods [5,265]. This section focuses on key genetic polymorphisms that have been investigated in relation to strength athletic performance. Specifically, attention will be given to variants in the arkadia (RNF111) N-terminal like PKA signaling regulator 2N (*ARK2N/C18ORF25*), androgen receptor (*AR*), peroxisome proliferator-activated receptor gamma (*PPARG*), MMS22 like (*MMS22L*), leucine-rich pentatricopeptide repeat containing (*LRPPRC*), phosphatase and actin regulator 1 (*PHACTR1*) and methylenetetrahydrofolate reductase (*MTHFR*) genes. These polymorphisms are implicated in molecular pathways related to muscle hypertrophy, androgen signaling, energy utilization, and cytoskeletal dynamics, all of which contribute to the development and optimization of strength-related phenotypes in elite athletes.

### 5.1. Arkadia (RNF111) N-Terminal Like PKA Signaling Regulator 2N (ARK2N/C18ORF25)

*ARK2N*, also known as *C18ORF25*, encodes a protein called Arkadia N-terminal-like PKA signaling regulator 2N, which plays a role in cellular signaling pathways and calcium regulation within muscle fibers. These functions are essential for effective muscle contraction and optimal force output, making the gene a significant contributor to muscle physiology, particularly in the context of adaptations to strength-based training [266,267,268]. Recent research by Çığırtaş et al. [269] demonstrated that ARK2N expression is considerably higher in strength athletes compared to endurance athletes, underscoring its relevance in oxidative fast-twitch muscle fibers (type IIA), which are prevalent in strength sports [270]. A noteworthy genetic variation within this gene is the rs6507691 (C/T) polymorphism. The T allele has been linked to increased gene expression and a larger cross-sectional area of muscle fibers. Athletes with this allele tend to exhibit greater muscle fiber size, both in fast and slow types, translating to superior strength and power capabilities [271,272]. This T allele is significantly more frequent among elite strength athletes, suggesting a genetic edge in muscle performance [269]. Additionally, since this polymorphism acts as an expression quantitative trait locus (eQTL), it affects ARK2N transcription levels. This variation supports muscle hypertrophy and enhances contraction under load via its role in calcium signaling, a key mechanism for explosive muscular action [273].

### 5.2. Androgen Receptor (AR)

Androgens are steroid hormones synthesized from cholesterol that influence not only reproductive organs but also various other tissues, including skeletal muscle, where they play a key anabolic role [274]. Their synthesis begins with the conversion of cholesterol into pregnenolone, initiating a cascade of biochemical reactions that lead to the production of androstenedione, a major precursor of testosterone. Testosterone, together with more potent metabolites such as dihydrotestosterone (DHT), binds to the androgen receptor, especially in muscle tissue, promoting proteins synthesis and muscle growth [275,276]. At the molecular level, the androgen receptor protein comprises three functional domains: a COOH-terminal ligand-binding domain, a central DNA-binding domain, and an NH_2_-terminal domain responsible for transcriptional activation via ligand-dependent and protein–protein interactions [277]. The androgen receptor (*AR*) gene, located on the long arm of the X chromosome, includes a polymorphic region in exon 1 composed of CAG trinucleotide repeats, typically ranging between 8 and 37 repeats [278]. These repeats encode a polyglutamine (polyQ) tract in the N-terminal domain, and the length of this tract can modulate intra-receptor interactions, particularly N/C-terminal communication, a key step in receptor dimerization and transcriptional activation [279]. When AR binds DNA, this N/C interaction is disrupted, allowing the recruitment of transcriptional coactivators [280]. It has been suggested that longer polyQ tracts may hinder this process by reducing coactivator recruitment, thereby impairing transcriptional activity [281]. Conversely, shorter CAG sequences, despite being associated with lower basal transcriptional activity, might negatively affect cellular differentiation and muscle strength [282,283]. Empirical studies investigating the relationship between CAG repeat length and muscle phenotypes report mixed findings. For instance, Walsh et al. observed that Caucasian males with 22 CAG repeats exhibited greater lean body mass than those with fewer [284]. Likewise, Campbell et al., studying the Ariaal population in Kenya, found a positive association between CAG length and lean mass [285]. Conversely, Nielsen et al. reported a negative correlation between repeat number and muscle area in young Danish men [286]. Further evidence by Guilherme et al. demonstrated that bodybuilders with more than 21 CAG repeats had higher muscle strength and mass, both in upper and lower limbs. This genotype was also more prevalent among elite power athletes such as sprinters, weightlifters, and bodybuilders, suggesting a possible optimal CAG range that facilitates muscular adaptation to strength training [287]. In line with these observations, Morton et al. found that androgen receptor content in muscle was a key determinant of hypertrophic response to resistance training. In so-called “high responders”, AR levels were significantly elevated and positively correlated with muscle mass gains [288,289]. This supports the notion that AR-regulated genes are central to skeletal muscle growth and adaptation, and that AR signaling plays a crucial role in exercise-induced hypertrophy [290,291]. Beyond muscle function, the *AR* (CAG)n polymorphism also impacts glucose metabolism and insulin sensitivity. A higher number of CAG repeats is linked to lower AR transcriptional activity, which may negatively affect glucose homeostasis and insulin action, increasing the risk of type 2 diabetes [292,293]. This may involve altered regulation of glucose-related genes, β-cell function, and fat distribution, all contributing to insulin resistance [294]. Therefore, evaluating CAG repeat length might be useful in assessing androgenic function and metabolic risk, especially in men with type 2 diabetes [295].

### 5.3. Peroxisome Proliferator-Activated Receptor Gamma (PPARG)

The peroxisome proliferator-activated receptor gamma (*PPARG*) gene, located on chromosome 3p25, encodes PPARγ, a ligand-activated nuclear transcription factor that belongs to the nuclear receptor superfamily. PPARγ plays a critical role in the regulation of adipocyte differentiation, lipid storage, insulin sensitivity, and glucose metabolism by modulating the expression of genes involved in lipid and carbohydrate pathways [296]. The *PPARG* gene undergoes alternative promoter usage and alternative splicing, resulting in the generation of multiple mRNA isoforms with tissue-specific expression patterns [297]. Among these, the PPARγ2 isoform is distinguished by the presence of an additional 28 amino acids at its N-terminal region, encoded by exon B [298]. A common SNP in exon B, rs1801282 (C/G), results in a Pro12Ala amino acid substitution in the PPARγ2 protein [299]. The 12Ala variant exhibits reduced binding affinity for peroxisome proliferator response elements (PPREs) in target gene promoters, resulting in attenuated transcriptional activity [300,301]. Despite this, the 12Ala allele has been associated with improved insulin sensitivity and enhanced glucose uptake in skeletal muscle [302], physiological traits that may confer an advantage in short-duration, high-intensity athletic activities such as sprinting, throwing, and weightlifting. Supporting this hypothesis, Ahmetov et al. reported that carriers of the 12Ala allele exhibited a significantly greater cross-sectional area (CSA) of type I (slow twitch) muscle fibers compared to Pro12 homozygotes [303]. Similar findings were reported by Maciejewska-Karlowska et al., who observed a significantly higher frequency of the 12Ala allele among Polish athletes engaged in strength- and power-oriented disciplines, suggesting a selective advantage for anaerobic performance traits [304]. However, the 12Ala allele has also been associated with unfavorable metabolic profiles in some populations, including elevated total cholesterol (TC), increased body mass index (BMI), and greater waist circumference (WC) when compared to Pro12Pro homozygotes. Notably, the phenotypic expression of this polymorphism appears to be modulated by variables such as ethnicity, lifestyle, sex, and age, which may influence its metabolic and performance-related effects [305].

### 5.4. MMS22 Like (MMS22L), Leucine-Rich Pentatricopeptide Repeat-Containing (LRPPRC), Phosphatase and Actin Regulator 1 (PHACTR1) and Methylenetetrahydrofolate Reductase (MTHFR)

Recent studies have identified specific genetic variants potentially associated with enhanced strength performance, highlighting an emerging area of interest in sports genomics that warrants further exploration [306]. Among these, the MMS22 like (*MMS22L*) gene, which encodes a protein involved in DNA repair mechanisms, has gained attention for its role in maintaining genomic integrity during and following high-intensity physical activity [307]. The rs9320823 (T/C) polymorphism in *MMS22L* gene, particularly T allele, has been linked to increased muscular strength, suggesting that carriers may possess superior recovery and muscle adaptation capacities, traits critical for success in power-based sports such as weightlifting [306,308]. Similarly, the leucine-rich pentatricopeptide repeat-containing (*LRPPRC*) gene, which regulates mitochondrial transcription and contributes to cytoskeletal organization, appears to be relevant to muscle performance [309]. The rs10186876 (A/G) polymorphism in *LRPPRC* gene, particularly A allele, has been associated with elevated gene expression in skeletal muscle, implying a potential enhancement in contractile force generation during resistance exercise [306,308]. Furthermore, the phosphatase and actin regulator 1 (*PHACTR1*) gene also plays a key role in cytoskeletal dynamics, which are essential for muscle contraction and cellular mobility [310]. The rs6905419 (C/T) polymorphism in *PHACTR1* gene, particularly the C allele, has been identified as a marker of muscular strength, with carriers exhibiting superior performance outcomes compared to non-carriers [306,308]. Finally, the methylenetetrahydrofolate reductase (*MTHFR*) gene, which is integral to folate metabolism and DNA methylation, may also influence athletic performance [311]. The rs1801131 (A/C) polymorphism in the *MTHFR* gene, particularly the C allele, has been associated with enhanced athletic performance, potentially due to improved energy metabolism and more effective oxidative stress regulation during high-intensity exercise [306,308,312]. Beyond its implications in sports, this polymorphism has also been linked to metabolic risk factors. Specifically, Poodineh et al. demonstrated that carriers of the C allele exhibited an increased risk of type 2 diabetes (T2D) onset and progression [313]. Supporting this evidence, Yan et al. reported a similar association between rs1801131 and elevated diabetes risk in a Chinese population [314]. Furthermore, Zhou et al. found that individuals with the CC genotype had a significantly higher risk of ischemic stroke, suggesting that *MTHFR* polymorphisms may modulate susceptibility to various disorders by affecting total homocysteine concentrations and MTHFR enzymatic activity [315].

## 6. Genes Associated with Injuries

The capacity to sustain training over time without incurring injury is a fundamental component of athletic performance, alongside endurance, strength, and technical proficiency. Injuries can disrupt training cycles, hinder performance progression, and compromise competitive success. While extrinsic factors, such as training volume, load, and technique are traditionally recognized as primary contributors to injury risk, increasing evidence suggests that genetic predisposition also plays a critical role in determining individual susceptibility to musculoskeletal injuries [316]; for example, the genetic contribution to anterior cruciate ligament (ACL) rupture has been estimated at ~69%, highlighting the potential impact of familial and genetic factors on injury risk [317]. Among the most studied genetic determinants are polymorphisms in genes encoding for collagen, a key structural protein that contributes to the mechanical strength and integrity of connective tissues. Variants in collagen-related genes have been linked to a greater risk of spontaneous or non-contact soft-tissue injuries, particularly in high-demand sports contexts [318]. However, injury susceptibility is not solely attributable to structural components. Inflammatory and reparative processes, central to tissue recovery following mechanical stress, are also regulated at the genetic level. In this regard, cytokines, including interleukins, interferons, chemokines, growth factors, tumor necrosis factors, and adipokines, represent a class of signaling molecules essential to the coordination of immune and regenerative responses [319]. Several genes have been identified as potential determinants of injury susceptibility, including collagen type I alpha 1 chain (*COL1A1*), collagen type V alpha 1 chain (*COL5A1*), interleukin 6 (*IL6*), vascular endothelial growth factor A (*VEGFA*) and noggin (*NOG*). These genes are involved in key processes, such as collagen synthesis, inflammation regulation, and angiogenesis, influencing connective tissue strength and the ability to recover from biomechanical stress.

### 6.1. Collagen Type I Alpha 1 Chain (COL1A1) and Collagen Type V Alpha 1 Chain (COL5A1)

Collagen type I alpha 1 chain (*COL1A1*) and collagen type V alpha 1 chain (*COL5A1*) encode the α-chains of type I and type V collagen, respectively, essential structural components of the extracellular matrix that play a central role in maintaining the mechanical integrity of connective tissues, particularly in bones, tendons, and ligaments [320]. *COL1A1*, located on chromosome 17q21.33, encodes the α1 chain of type I collagen, the most abundant collagen in the human body. This triple-helical structure, composed of two α1 chains (*COL1A1*) and one α2 chain (*COL1A2*), provides high tensile strength in skeletal tissues such as bone, tendon, skin, and dentin [321]. Conversely, *COL5A1*, located on chromosome 9q34.3, encodes the α1 chain of type V collagen which, though less abundant, is crucial for regulating type I collagen fibril assembly and morphology [322]. Mutations or polymorphisms in these genes have been associated with a range of connective tissue disorders, including Ehlers-Danlos syndrome [323], osteogenesis imperfecta [324], and an increased risk of musculoskeletal injuries such as tendon and ligament ruptures [325,326]. The rs1800012 G/T), also known as the Sp1 polymorphism, in *COL1A1* lies within the first intron at a binding site for the Sp1 transcription factor [327]. Although its precise effect on gene expression remains unclear [328], recent meta-analytic evidence suggests that the TT genotype may confer a protective effect against sport-related soft tissue injuries, potentially by enhancing the mechanical resilience of connective structures under high strain [329]. Another variant of interest, the rs1107946 (G/T), may play a role in bone mineralization [330], although its relevance to injury susceptibility is yet to be fully elucidated [331]. In the case of *COL5A1*, this gene contributes to the regulation of fibrillar architecture by limiting lateral fibril growth, thus affecting collagen organization in tendons and ligaments [332,333]. Certain polymorphisms in the 3′-UTR, particularly the rs12722 (C/T), are believed to alter mRNA stability and gene expression levels [334]. Studies involving athletes have linked the TT genotype to a higher risk or severity of musculoskeletal injuries compared to CC carriers [335]. Nonetheless, findings are mixed; while research on Japanese cohorts has not supported an association between the rs12722 (C/T) and passive muscle stiffness or injury risk [336], a recent meta-analysis confirmed a significant correlation between this variant and ligament injury susceptibility, particularly in Caucasian populations [337]. Furthermore, rs12722 has also been implicated in the pathogenesis of chronic Achilles’ tendinopathy [333].

### 6.2. Interleukin 6 (IL6)

Interleukin 6 (*IL6*) gene encode for interleukin-6 (IL-6), a multifunctional cytokine that regulates inflammation, metabolic processes, and tissue repair through its receptor IL-6R, encoded by the *IL6R* gene. Plasma levels of IL-6 increase significantly during exercise, depending on factors such as intensity, duration, and the amount of muscle mass recruited [338]. IL-6 also plays a role in triggering the acute-phase response and antibody production [319,339]. Following injury, particularly to tendons or ligaments, IL-6 is released by fibroblasts and participates in immune regulation, inflammation, and hematopoiesis [340]. It also supports muscle regeneration by promoting myoblast activity and may aid tendon healing. With both pro- and anti-inflammatory roles, IL-6 can contribute to the transition from acute to chronic inflammation [319,340], and its levels peak in the synovial fluid a few days after anterior cruciate ligament (ACL) injury, indicating involvement in early healing stages [341]. Additionally, IL-6 influences bone resorption, apoptosis, and collagen production [342], and its secretion increases under mechanical stress in tendon cells, which may reflect pathological responses in connective tissues [343]. Several *IL6* gene polymorphisms have been studied in relation to injury susceptibility, particularly due to their role in inflammation and tissue repair. The GG genotype of the *IL6* rs1800795 (G/C) polymorphism has been linked to a 1.68-fold higher risk of hamstring injuries compared to the GC and CC genotypes [344]. The G allele has been shown to enhance *IL6* gene expression and increase plasma IL-6 levels in response to stress stimuli [345], and has been previously associated with Achilles’s tendinopathy [346], lumbar disc degeneration [347], and power/strength athlete status [62]. On the other hand, the CC genotype has been associated with higher creatine kinase levels following eccentric exercise in healthy individuals [342]. Although *IL6* gene variants have been studied in relation to various diseases, their association with susceptibility to rotator cuff tears (RCT) is not well understood. Two polymorphisms, the rs1800795 (G > C) and the rs1800797 (A > G), located in the promoter region of the IL6 gene, influence the production of IL-6 in plasma [348,349]. The rs1800795 polymorphism has been significantly linked to an increased risk of RCT, particularly in homozygous and allelic models. Furthermore, the effect of the rs1800797 polymorphism on RCT risk is more pronounced in women, individuals who consume alcohol, and those with a BMI less than 25 kg/m^2^ [350]. Other research has shown that polymorphisms in these genes can contribute to the risk of musculoskeletal injuries. For example, the *IL6* rs1800795 polymorphism has been significantly associated with ACL rupture risk [351]. The interaction between IL-6 and IL-6R is critical, as the soluble form of IL-6R affects the activity of IL-6, modulating the inflammation and tissue healing process [352]. However, receptor variants, like *IL6R* rs2228145 (A/C), did not show direct associations with ACL injuries in some studies, but remain important candidates for future research [353]. Combinations of these genetic variants may also influence injury risk by altering the inflammatory response and matrix remodeling [346,351].

### 6.3. Vascular Endothelial Growth Factor A (VEGFA)

Angiogenesis-associated signaling pathways have also been explored in relation to various orthopedic conditions [354]. More recently, genetic variations in genes involved in these pathways have been linked to increased susceptibility to anterior cruciate ligament (ACL) injuries [355]. A key regulator of these signaling pathways is the vascular endothelial growth factor (*VEGF*) gene, located on chromosome 6 at position 6p21.1, which encodes the VEGF protein [356,357]. The gene spans approximately 14 kb and comprises a coding region with 8 exons and 7 introns [358] and includes over 30 documented SNPs across five isoforms: *VEGF-A*, *VEGF-B*, *VEGF-C*, *VEGF-D*, and placental growth factor [359]. Notably, the *VEGFA* promoter SNP rs699947 (C > A) has been implicated in a range of pathological conditions, including coronary artery disease, cancer, rheumatoid arthritis, and several forms of tendinopathy [355,358,360,361]. Consequently, multiple studies suggest that genetic variations within the *VEGFA* gene may be associated with an increased risk of tendon or ligament injuries [362,363]. The promoter region of VEGFA contains several common SNPs that play a regulatory role in controlling gene expression. Notable, among these are −2578C/A (rs699947), −1154G/A (rs1570360), −634C/G (rs2010963), and −2549 I/D (rs35569394). In the European population, individuals with AA or AC genotypes of rs699947 (dominant model) showed a significantly lower risk of tendon and ligament injuries compared to those with the CC genotype [364]. Likewise, the AG genotype of rs1570360, (over-dominant model) was found to provide a protective effect [365]. Interestingly, the rs1570360 GG genotype is associated with increased VEGFA expression [363]. However, excessive VEGFA expression may negatively affect biomechanical tendon strength, potentially raising injury risk. Moreover, individuals with the GG genotype also tend to have higher body weight, which may further contribute to tendon and ligament injury susceptibility [366]. In terms of the rs2010963 polymorphism, the G allele appears to confer a protective effect, with those carrying the GG genotype in the additive model showing a lower likelihood of such injuries [365]. The −634C > G polymorphism (rs2010963) in homozygosity (GG) has been linked to reduced VEGF expression under hypoxic conditions, which in turn decreases maximal oxygen consumption (VO_2_ Max) [367]. However, the same study showed that in heterozygosity (CG), VEGF expression under hypoxia is null, but VO_2_ Max remains unaffected. In an analysis of 30 soccer players, the most frequent genotype was CG (46%), followed by GG (32%) and CC (22%) [368]. Additionally, a study on 670 Russian athletes revealed that the C allele is associated with better aerobic performance and more efficient lactic acid metabolism [369]. In soccer players, a significant prevalence of the CC homozygote was observed, correlating with superior aerobic capacities, indirectly confirming the association between the G-634C polymorphism and physical performance [368]. A recent study found that the *VEGFA* rs699947 A allele and CA genotype were significantly more frequent in Indian athletes with ACL injuries, especially in non-contact cases, indicating a 2.75–3.23 times higher risk compared to controls [364]. These findings contrast with results from African populations, suggesting ethnic genetic differences [370]. Haplotype analysis showed complete linkage disequilibrium between *VEGFA* rs699947 (-2578 C/A) and rs35569394 (-2549 18bp I/D) [364]. The C-D haplotype, linked to higher VEGF expression, was more common in the control group, suggesting a protective role in tissue healing and ACL strength. In contrast, the A-I haplotype was more frequent in individuals with ACL injuries [364]. A recent meta-analysis indicated that the *VEGFA* rs699947 C allele is linked to a lower risk of tendon and ligament injuries in athletes, though the studies showed some variability. Further research is needed to better understand the genetic profiles of athletes prone to such injuries [371].

### 6.4. Noggin (NOG)

Noggin (*NOG*) gene, located on chromosome 17q22, encodes Noggin, a small, secreted glycoprotein [372]. This protein plays a pivotal role during embryonic development by antagonizing bone morphogenetic proteins (BMPs), which are members of the transforming growth factor-beta (TGF-β) superfamily involved in regulating cellular proliferation, differentiation, and apoptosis [373]. Inhibiting BMP signaling, Noggin ensures proper formation of the nervous system, skeletal structures, and mesenchymal stem cell differentiation during embryogenesis [374]. Alterations in *NOG* expression have been linked to various congenital skeletal abnormalities, including tarsal-carpal coalition and other bone malformations [375]. In the context of bone repair, Noggin also plays a regulatory role: BMPs stimulate the proliferation and differentiation of mesenchymal stem cells (MSCs) and osteoprogenitor cells [376,377], but their activity is tightly controlled by endogenous inhibitors. Among these, Noggin functions as a key extracellular antagonist, preventing BMPs from binding to their receptors [378,379]. Genetic variations affecting Noggin function may disrupt BMP signaling and interfere with growth differentiation factor (GDF) activity. Notably, the rs1372857 SNP in the *NOG* gene has been associated with an increased risk of atrophic non-union, particularly in individuals carrying the GG genotype [380]. Supporting this, Jacob et al. reported a higher incidence and severity of muscle injuries among Australian football players carrying the same genotype [381]. These findings suggest a potential role for Noggin in muscle-tendon-bone biomechanics, highlighting the need for further investigation into its contribution to injury susceptibility.

## 7. Genes Predisposing Individuals to Metabolic Risk Modulated by Physical Exercise

It is increasingly recognized that, just as gene expression can influence physical performance, physical activity itself can modulate gene expression, potentially counteracting the detrimental effects of specific polymorphisms [382]. In this context, three genes have been extensively investigated for their contribution to metabolic risk: FTO alpha-ketoglutarate-dependent dioxygenase (*FTO*), peroxisome proliferator-activated receptor gamma (*PPARG*), and adrenoceptor beta 3 (*ADRB3*). These genes are involved in the regulation of energy metabolism and insulin sensitivity, and their polymorphic variants have been associated with an increased susceptibility to metabolic disorders. However, evidence indicates that physical activity plays a crucial compensatory role, attenuating genetic predisposition to these conditions.

### 7.1. FTO Alpha-Ketoglutarate-Dependent Dioxygenase (FTO)

The FTO alpha-ketoglutarate-dependent dioxygenase (*FTO*) gene, located on chromosome 16q12.2, has emerged as a major genetic determinant of obesity, with variants in intron 1 being strongly linked to increased body mass index (BMI) and obesity risk [383,384]. Among these, the rs9939609 polymorphism is one of the most studied. Individuals homozygous for the risk allele A (AA) exhibit a ~1.7-fold higher likelihood of developing obesity compared to TT carriers [385]. This increased susceptibility is partly attributed to altered appetite regulation, reduced satiety, and dysregulated hormonal responses involving the ghrelin-appetite axis [386,387]. Karra et al. reported an attenuated suppression of acylated ghrelin (AG), a key orexigenic hormone, in AA individuals, suggesting a potential mechanism behind increased food intake [388]. Physical exercise has been identified as a potent modulator of the adverse effects associated with FTO polymorphisms. Acute bouts of moderate-to-high intensity exercise reduce circulating AG levels while increasing anorexigenic hormones such as PYY and GLP-1, contributing to a temporary negative energy balance [389,390,391]. Notably, in AA carriers, a single exercise session has been shown to increase the activity of butyrylcholinesterase (BChE), an enzyme that hydrolyzes AG to desacyl-ghrelin (DAG), which may have appetite-suppressing properties, thereby reducing the AG:DAG ratio, a key indicator of energy intake regulation [392,393,394,395]. Dorling et al. demonstrated that AA individuals exhibit lower fasting BChE activity and a higher postprandial AG:DAG ratio compared to TT carriers, corresponding to increased appetite and energy consumption [396]. However, these differences were eliminated following physical activity, indicating a genotype-compensatory effect induced by exercise [396,397,398]. At the molecular level, *FTO* may function as an “energy sensor” in metabolic tissues, especially in skeletal muscle, where it modulates metabolic pathways in response to substrate availability and energetic demands [399,400]. Acute high-intensity exercise has been shown to significantly reduce *FTO* mRNA expression in skeletal muscle, particularly in AA carriers, suggesting a genotype-specific transcriptional response to physical activity [401]. Overall, this evidence supports the hypothesis that physical activity directly modulates molecular pathways altered by the *FTO* risk allele, thereby reducing obesity risk in genetically predisposed individuals. Numerous studies have confirmed the interaction between the *FTO* genotype and an active lifestyle, with physical activity shown to attenuate the effect of the rs9939609 A allele on BMI by up to 30–47% [398,402], as well as reduce the risk of associated comorbidities such as type 2 diabetes, hypertension, and all-cause mortality [402,403,404]. These findings underscore the protective role of exercise in the gene–environment interaction and highlight its importance in the prevention and management of obesity in genetically susceptible individuals.

### 7.2. Peroxisome Proliferator-Activated Receptor Gamma (PPARG)

The peroxisome proliferator-activated receptor gamma (*PPARG*) gene encodes the peroxisome proliferator-activated receptor gamma (PPARγ), which is predominantly expressed in adipose tissue, colon, and macrophages. It plays a crucial role in glucose homeostasis, lipid storage, and cardiovascular metabolism [300,405,406]. Located on chromosome 3p25.2, one of its most widely studied SNP is rs1801282 (C/G) (Pro12Ala), which leads to an amino acid substitution that decreases the receptor’s transcriptional activity. Among Caucasian populations, the Ala variant has been initially associated with lower body mass index (BMI) and improved insulin sensitivity, especially in individuals with obesity or type 2 diabetes (T2D) [407,408,409,410]. However, these associations are not uniform and appear to be influenced by environmental factors such as diet, gender, and physical activity levels [411]. Intervention studies have shown that physical exercise can mitigate or even reverse the metabolic risks conferred by adverse genotypes. Notably, Ala carriers demonstrate enhanced metabolic responsiveness to aerobic training compared to Pro/Pro homozygotes, exhibiting greater improvements in peripheral insulin sensitivity, glucose tolerance, and pancreatic β-cell function, evidenced by increased acute insulin response and disposition index [412,413]. This positive effect is primarily attributed to faster skeletal muscle glucose disposal rather than differences in insulin clearance or hepatic glucose production. Additionally, Ala carriers show increased glucose effectiveness, i.e., the ability of glucose itself to promote uptake and suppress endogenous production, independently of insulin levels [412]. These benefits have been observed across various demographic and clinical contexts, including individuals at high risk of T2D or with a family history of the disease [413,414]. Although the Ala allele is generally considered protective, in individuals with impaired glucose tolerance (IGT) and low BMI, it may be linked to an increased risk of developing T2D, likely due to reduced insulin secretion capacity [415]. Nevertheless, higher physical activity levels have been shown to negate this adverse effect, reinforcing the importance of gene–environment interactions [414]. Adipose tissue lipid metabolism may be affected by the Pro12Ala variant, which promotes insulin-driven suppression of lipolysis and lowers non-esterified fatty acids (NEFA) concentrations, facilitating glucose usage in skeletal muscle through the Randle cycle [416,417,418]. Furthermore, physical activity appears to increase adiponectin expression in Ala carriers, contributing to their improved insulin sensitivity [419]. In summary, physical exercise serves as a powerful modulator of the phenotypic expression of the *PPARG* Pro12Ala polymorphism, reducing the likelihood of T2D onset and improving metabolic parameters, particularly in Ala carriers. Nonetheless, Pro/Pro individuals also benefit from exercise, albeit to a lesser extent, highlighting the universal importance of lifestyle interventions in preventing metabolic disorders.

### 7.3. Adrenoceptor Beta 3 (ADRB3)

The adrenergic system plays a central role in energy balance regulation, primarily through thermogenesis in brown adipose tissue and lipolysis in white adipose tissue across humans and other species [420]. The adrenoceptor beta 3 (*ADRB3*) gene, located on chromosome 8p11.23, encodes the β3-adrenergic receptor, a protein predominantly expressed in visceral and subcutaneous adipose tissue, where it mediates catecholamine-induced lipolysis and thermogenesis [421,422].One of the most extensively studied variant in this gene is the Trp64Arg polymorphism (rs4994), which results from a thymine-to-cytosine substitution (T > C) leading to an arginine instead of a tryptophan at position 64 in the receptor’s first intracellular loop [423]. Carriers of the Arg64 allele exhibit increased resistance to weight loss and a diminished reduction in visceral fat, along with a higher risk of lipid abnormalities, obesity, type 2 diabetes mellitus (T2D), and impaired fat oxidation [422,424]. In adolescents, Jesus et al. found that Arg64 carriers had elevated LDL-c levels, indicating a potentially greater future cardiovascular risk [425]. Similarly, in lean Japanese adults, the Arg64Arg genotype was significantly correlated with LDL-c levels and age, showing an annual increase in BMI [426]. Conversely, a study in non-obese Italian adults did not report a clear effect of Arg64 on lipid profiles but did link it to increased abdominal adiposity [427]. Among obese individuals, those with the Trp64Arg genotype tend to gain more weight compared to Trp64Trp homozygotes [428]. The association between this variant and insulin resistance remains inconsistent across adult and pediatric populations [429,430]. Nonetheless, in overweight adolescents, Arg64 carriage was linked to insulin resistance, though improvements in body composition and fitness were achieved regardless of genotype following aerobic training and nutritional counseling [431]. A further study in healthy Japanese subjects demonstrated that Arg64 homozygosity was associated with greater carotid intima-media thickness (ccIMT), a marker of atherosclerotic risk, but only among those with poor cardiorespiratory fitness. In contrast, no such association was observed in fit individuals, suggesting that aerobic fitness may buffer the cardiovascular risk linked to this genotype [432]. Finally, a case–control study confirmed that the obesity risk associated with the Trp64Arg variant is modulated by levels of physical activity: sedentary Arg64 carriers exhibited a stronger predisposition to weight gain, whereas this effect was significantly attenuated in physically active individuals [433]. These findings support the idea that genetic susceptibility can be counterbalanced by an active lifestyle, and that genotyping may help tailor preventive strategies.

## 8. Discussion

The expanding field of sports genomics offers promising perspectives for both optimizing athletic performances and personalizing clinical exercise interventions [2]. This review highlights how genetic polymorphisms in key genes influence not only sport-specific phenotypes such as endurance, power, and strength, but also modulate injury risk and predisposition to metabolic disorders (Table 1). These aspects are increasingly relevant in the context of precision medicine, where individual genomic profiles may inform tailored approaches to training, rehabilitation, and chronic diseases prevention [2,4].

In particular, in clinical context, the assessment of performance-related gene variants represents a novel strategy to enhance the personalization of physical activity prescriptions. Genetic screening could support clinicians in identifying individuals predisposed to metabolic impairment, musculoskeletal injury, or poor adaptation to standard training protocols. For instance, the identification of *FTO* or *PPARG* risk alleles could guide early lifestyle interventions in individuals at high risk for obesity or type 2 diabetes [402,403,413], while variants in *COL1A1* or *IL6* may allow the identification of increased susceptibility to tendon or ligament injuries [329,350,362]. Such information could be used to optimize exercise modalities, intensities, and recovery plans, especially in rehabilitation settings or for patients with chronic cardiometabolic conditions [15,33].

Based on all these considerations, commercial direct-to-consumer genetic tests currently offer analysis of SNPs panels. Although their primary market remains sport performance, their potential clinical utility is currently under evaluation. Some models, such as the Total Genotype Score (TGS), attempt to integrate multiple loci into predictive frameworks for athletic potential or exercise responsiveness [19]. Despite the great potential, these tools still lack extensive validation in clinical populations, and their predictive power remains limited.

Indeed, despite the great potential for future applications in different clinical settings, several limitations constrain the clinical applicability of exercise genomics. First, many reported associations are based on the analysis of relatively small, ethnically homogeneous cohorts, and are rarely replicated in large-scale, multi-ethnic populations. The effect size of most individual variants is modest. Many of these variants exhibit low penetrance and are classified in databases such as ClinVar as benign or of uncertain significance. Consequently, their contribution to phenotypic traits is limited, context-dependent, and often insufficient for reliable prediction in athletic or clinical settings. In addition gene–environment interactions, including training history, nutrition, and psychological factors, significantly modulate phenotypic expression [3,12,21]. Moreover, there are no standardized guidelines on how to interpret or act upon genetic findings in the context of exercise therapy, and results often lack clear thresholds for decision-making. The classification of low-penetrance variants and variants of uncertain significance further complicates the translation of genetic data into actionable recommendations, emphasizing the need for rigorous criteria and validation before clinical implementation [434].

Furthermore, additional large-scale genome-wide association studies (GWAS) are needed to validate reported associations, identify novel variants, and advance the field of exercise genomics.

In addition, ethical and practical concerns must be addressed. The potential for genetic discrimination, overinterpretation of risk, and the propagation of genetic determinism must be carefully managed, particularly in vulnerable populations, such as children or patients with chronic disease. Any implementation of genetic testing in clinical or athletic settings should be accompanied by appropriate genetic counseling and a multidisciplinary framework that integrates molecular, physiological, and behavioral data.

Future studies should focus on validating polygenic models in diverse populations, assessing longitudinal outcomes, and developing evidence-based protocols that translate genomic data into actionable strategies. A robust, interdisciplinary approach is essential to fully realize the potential of personalized exercise medicine.

## 9. Conclusions

Data presented in this review emphasize the relevance of genetic variability in influencing physical performances, injury risk, and susceptibility to metabolic diseases. The identification of specific polymorphisms associated with endurance, power, and strength phenotypes underscores the importance of considering the individual’s genetic makeup in the development of tailored training protocols, aimed not only at enhancing athletic performance but also at promoting long-term health. Furthermore, the overlap between certain performance-related genes and those implicated in chronic metabolic conditions suggests that exercise can serve as a powerful modulator of genetically mediated disease risk. In particular, physical activity has been shown to mitigate the effects of risk alleles associated with obesity and type 2 diabetes mellitus, highlighting its preventive and therapeutic potential. The genetic approach discussed here may hold promise on neurodevelopmental or neurodegenerative disorders, given the growing evidence supporting the role of exercise in modulating disease progression and improving neurological outcomes. Importantly, the practical and ethical implications of integrating genetic testing into training or rehabilitation programs should be carefully considered. Applications should be accompanied by appropriate genetic counseling and a multidisciplinary framework to ensure responsible use, avoid overinterpretation of risk, and prevent potential discrimination, especially in vulnerable populations such as children or patients with chronic diseases.

Future research should focus on the validation and refinement of polygenic or multi-trait genetic panels, including assessment in diverse populations, longitudinal studies, and the development of evidence-based protocols to translate genomic data into actionable exercise interventions. These steps are essential to fully realize the potential of personalized exercise medicine while maintaining ethical and practical standards. Altogether, polymorphisms examined in this work could serve as a foundation for the future development of multi-trait genetic panels as diagnostic tools, supporting both athletic profiling and clinical decision-making. The integration of genetic data into sports science and preventive medicine represents a significant step toward truly personalized exercise interventions, tailored to optimize performance, reduce injury risk, and promote health across diverse populations.

## Figures and Tables

**Table 1:** Overview of the genes discussed in this review, summarizing genetic information (gene symbol, full name, chromosomal locus, polymorphism, predisposing allele, and allelic frequency) and associated phenotypic impact.

Gene	Full Name	Locus	Polymorphism	Predisposing Allele	Allelic Frequency (%)	Associated Phenotype
**Genetic Variants Associated with Endurance Performance**						
*ACE*	Angiotensin I converting enzyme	17q23.3	rs1799752 (I/D)	Alu I	I: 40% D: 60%	Enhanced endurance performance [55,56,57]
*PPARGC1A*	PPARG coactivator 1alpha	4p15.1	rs8192678 (G1444A)	G (Gly482)	G: 73% A: 27%	Greater oxidative capacity, higher mitochondrial content, and enhanced fatigue resistance [88]
*HFE*	Homeostatic iron regulator	6p21.3	rs1800562 (G845A); rs1799945 (C187G)	A (Cys282) G (Asp63)	G: 99% A: 1% C: 93% G: 7%	Enhanced intestinal iron absorption and improved aerobic capacity [101,102,103,107,109,110]
*UCP2*	Uncoupling protein 2	11q13.4	rs660339 (C164T)	T (Val55)	C: 58% T: 42%	Enhanced maximal oxygen uptake, improved exercise efficiency, metabolic efficiency and endurance performance [119,120,121]
*UCP3*	Uncoupling protein 3	11q13.4	rs1800849 (-55C/T)	T	C: 70% T: 30%	Enhanced aerobic potential [124,126]
*CDKN1A*	Cyclin-dependent kinase inhibitor 1a	6p21.2	rs236448 (A/C)	A	A: 74% C: 26%	Enhanced endurance capacity and superior performance in aerobic-based sports [146]
*PPARA*	Peroxisome proliferator-activated receptor alpha	22q13.31	rs4253778 (G/C)	G	G: 73% C: 27%	Higher maximal aerobic capacity and enhanced oxygen pulse [159]
**Genetic Variants Associated with Power Performance**						
*ACTN3*	Actinin alpha 3	11q13.1	rs1815739 (C1729T)	C (Arg577)	C: 60% T: 40%	Enhanced muscle hypertrophy and power [178,180]
*SLC16A1/* *MCT1*	Solute carrier family 16 member 1	1p13.2	rs1049434 (A1470T)	T	A: 32% T: 68%	Increased anaerobic energy production [191]
*IGF1*	Insulin-like growth factor 1	12q23.2	rs35767 (C1245T)	T	C: 30% T: 70%	Improved power and endurance performance [215]
*AMPD1*	Adenosine monophosphate deaminase 1	1p13.3	rs17602729 (C34T)	C	C: 96% T: 4%	Increased anaerobic, high-intensity physical tasks [233]
*AGT*	Angiotensinogen	1q42.2	rs699 (T4072C)	C	T: 29% C: 71%	Higher levels of Ang II with increased power [243,244]
*AGTR2*	Angiotensin II receptor type 2	Xq23	rs11091046 (C3123A)	A	C: 53% A: 47%	Higher percentage of fast-twitch fibers and increased power [260]
**Genetic Variants Associated with Strength Performance**						
*ARK2N/C18ORF25*	*Arkadia (RNF111) N-terminal like PKA signaling regulator 2N*	4q13.3	rs6507691 (C/T)	T	C: 69% T: 31%	Enhanced muscle hypertrophy and contraction [273]
*AR*	Androgen receptor	Xq12	(CAG)n	CAG ≥ 21	~5–10%	Facilitated muscular adaptation to strength training [287]
*PPARG*	Peroxisome proliferator-activated receptor gamma	3p25	rs1801282 (C34G)	G	C: 93% G: 7%	Improved insulin sensitivity and enhanced glucose uptake in skeletal muscle [302]
*MMS22L*	MMS22 like	6q14.1	rs9320823 (T/C)	T	T: 31% C: 69%	Increased muscular strength [306,308]
*LRPPRC*	Leucine-rich pentatricopeptide repeat containing	2p21	rs10186876 (A/G)	A	A: 68% G: 32%	Enhancement in contractile force generation during resistance exercise [306,308]
*PHACTR1*	Phosphatase and actin regulator 1	6p24.1	rs6905419 (C/T)	C	C: 71% T: 29%	Increased muscular strength [306,308]
*MTHFR*	Methylenetetrahydrofolate reductase	1p36.22	rs1801131 (A/C)	C	A: 75% C: 25%	Improved energy metabolism and more effective oxidative stress regulation [306,308,312]
**Genetic Variants Associated with Injuries**						
*COL1A1*	Collagen type I alpha 1 chain	17q21.33	rs1800012 (+1245G/T); rs1107946 (-1997G/T)	T T	G: 91% T: 9% G: 26% T: 74%	Enhanced mechanical resilience of connective structures [329]
*COL5A1*	Collagen type V alpha 1 chain	9q34.2	rs12722 (C/T)	T	C: 65% T: 35%	Higher risk or severity of musculoskeletal injuries [335]
*IL6*	Interleukin 6	7p15.3	rs1800795 (G/C); rs1800797 (A/G)	G A	G: 86% C: 14% A: 14% G: 86%	Higher injuries risk [344]
*VEGFA*	Vascular endothelial growth factor A	6p21.1	rs699947 (−2578C/A); rs1570360 (−1154G/A); rs2010963 (−634C/G)	C G C	C: 68% A: 32% G: 81% A: 19% C: 33% G: 67%	Increased risk of tendon or ligament injuries [362,363,364,365,366,367]
*NOGGIN*	Noggin	17q22	rs1372857 (G/A)	G	G: 46% A: 54%	Higher incidence and severity of muscle injuries [381]
**Genetic Variants That Predispose to Metabolic Risk Mitigated by Physical Exercise**						
*FTO*	*FTO alpha-ketoglutarate-dependent dioxygenase (FTO)*	16q12.2	rs9939609 (T/A)	A	T: 66% A: 34%	Modulation of FTO molecular pathways reducing obesity risk in genetically predisposed individuals [398,402,403,404]
*PPARG*	Peroxisome proliferator-activated receptor gamma	3p25	rs1801282 (C34G)	C (Pro12)	C: 93% G: 7%	Enhanced metabolic responsiveness to aerobic training [412,413]
*ADRB3*	Adrenoceptor beta 3	8p12	rs4994 (T190C)	C (Arg64)	T: 88% C: 12%	Reduction in the cardiovascular risk linked to this genotype [432]

## Data Availability

No new data were created or analyzed in this study.

## Abbreviations

The following abbreviations are used in this manuscript:

TGS	Total Genotype Score
T2D	Type 2 Diabetes Mellitus
HbA1c	Glycated Hemoglobin
TNFα	Tumor Necrosis Factor-Alpha
IL-6	Interleukin-6
LDL	Low-Density Lipoprotein
HDL	High-Density Lipoprotein
BMI	Body Mass Index
ASD	Autism Spectrum Disorder
AD	Alzheimer’s Disease
PD	Parkinson Disease
SNPs	Single-Nucleotide Polymorphism
ACE	Angiotensin Converting Enzyme
PPARGC1A	Peroxisome Proliferator Activated receptor Gamma Coactivator 1-Alpha
HFE	Homeostatic Iron Regulator
UCP2	Uncoupling Protein 2
UCP3	Uncoupling Protein 3
CDKN1A	Cyclin-Dependent Kinase Inhibitor 1A
PPARA	Peroxisome Proliferator-Activated Receptor alpha
Ang II	Angiotensin II
RAAS	Renin–Angiotensin–Aldosterone System
Ang I	Angiotensin I
NO	Nitric Oxide
TFAM	Mitochondrial Transcription Factor A
MEF2	Myocite Enhancer Factor 2
ACTN3	Actinin Alpha 3
SLC16A1/MCT1	Solute Carrier Family 16 member 1
IGF-1	Insulin-like Growth Factor 1
AMPD1	Adenosine Monophosfate Deaminase 1
AGT	Angiotensinogen
AGTR2	Angiotensin II Receptor Type 2
MCT1	Monocarboxylate Transporter 1
CAD	Coronary Artery Disease
C18ORF25/ARK2N	Chromosome 18 Open Reading Frame 25
AR	Androgen Receptor
PPARG	Peroxisome Proliferator-Activated Receptor Gamma
MMS22L	Methyl Methanesulfonate Sensitivity Protein-22 Like
LRPPRC	Leucine-Rich Pentatricopeptide Repeat-Containing
PHACTR1	Phosphatase and Actin Regulator 1
MTHFR	Methylenetetrahydrofolate Reductase
COL1A1	Collagen Type 1 Alpha 1
COL5A1	Collagen Type V Alpha 1
VEGFA	Vascular Endothelial Growth Factor A
NOG	Noggin
FTO	Fat Mass and Obesity Associate
ADRB3	Adrenoreceptor Beta 3
ACL	Anterior Cruciate Ligament
